# Neuroinflammation and Perioperative Neurocognitive Disorders

**DOI:** 10.1213/ANE.0000000000004053

**Published:** 2019-03-19

**Authors:** Saraswathi Subramaniyan, Niccolò Terrando

**Affiliations:** From the Center for Translational Pain Medicine, Department of Anesthe siology, Duke University Medical Center, Durham, North Carolina.

## Abstract

Neuroinflammation has become a key hallmark of neurological complications including perioperative pathologies such as postoperative delirium and longer-lasting postoperative cognitive dysfunction. Dysregulated inflammation and neuronal injury are emerging from clinical studies as key features of perioperative neurocognitive disorders. These findings are paralleled by a growing body of preclinical investigations aimed at better understanding how surgery and anesthesia affect the central nervous system and possibly contribute to cognitive decline. Herein, we review the role of postoperative neuroinflammation and underlying mechanisms in immune-to-brain signaling after peripheral surgery.

Perioperative neurocognitive disorders, now encompassing acute delirium and longer-lasting postoperative cognitive dysfunction, are major challenges to our rapidly growing aging population that negatively affect cognitive domains such as memory, attention, and concentration after surgery.^1^ Patients who suffer from perioperative neurocognitive disorder are at risk for significant complications including dementia and even death.^2,3^ Although postoperative delirium has become *the most common* complication in older adults,^4^ the pathophysiology of these conditions remains unknown. Growing evidence suggests a possible role for neuroinflammation in this process because proinflammatory signaling molecules have been identified in both patients and animal models of perioperative neurocognitive disorder.

The aim of this review is to discuss recent evidence for the involvement of postoperative neuroinflammation in perioperative neurocognitive disorder, and to highlight possible mechanisms of relevance to perioperative neurocognitive disorder from preclinical and early clinical studies.

With continuous improvements in surgical technology and anesthesia care, increasingly sicker and older patients are exposed to often life-saving procedures. Unfortunately, many of these frail patients are left with postoperative delirium and longer-lasting cognitive decline, especially after cardiac and even noncardiac surgery. The incidence of delirium is estimated at 26%–53% and postoperative cognitive dysfunction at about 10% at 3 months.^5–7^ Even though perioperative neurocognitive disorder is observed in patients across different age groups and undergoing different surgical procedures, aging and operations such as cardiac and orthopedic surgery have become well-established risks factors for the development of these neurological complications.^8^ Recent clinical studies have used different approaches to show that both cardiac and noncardiac surgery trigger neuronal injury, which we briefly summarize below.

## BIOMARKERS

A recent study by Evered et al^9^ described a significant increase in plasma neurofilament light and tau, 2 key biomarkers classically associated with neuronal injury, as a result of exposure to general anesthesia and surgery. Several investigators have detected postoperative changes in Alzheimer’s disease biomarkers, including β-amyloid protein and intraneuronal neurofibrillary tangles (tau), in cerebrospinal fluid (CSF). Lower CSF β-amyloid protein/tau ratio has been associated with patients who develop perioperative neurocognitive disorder, suggesting a possible trajectory toward dementia after exposure to anesthesia and surgery.^10–12^ Changes in Alzheimer’s disease markers and astroglial cell integrity, as well as evidence for blood–brain barrier opening were also found in the CSF of patients after hip arthroplasty,^13^ confirming some of the earlier findings by Tang et al^14^ for idiopathic nasal CSF leak correction after surgery. Interestingly, although surgery modifies Alzheimer’s disease biomarkers and potentially accelerates their pathogenesis in some individuals, positron emission tomography imaging of β-amyloid protein plaque deposition has shown limited association with cognitive deficits 6 weeks after cardiac surgery.^15^ CSF and plasma inflammatory biomarkers, cytokines, and many other immune-soluble factors have been described over the past decade in response to different surgeries.^16–20^ Higher levels of CSF interleukin-6 were found to predict cognitive decline after coronary artery bypass surgery.^21^ Other proinflammatory markers such as C-reactive protein and interleukin-1β have also been linked to cognitive decline after cardiac procedures.^22^ Again, these changes in inflammatory markers are not limited to cardiac surgery and some of its unique aspects, for example, extracorporeal circulation and ischemia-reperfusion injury (reviewed in detail in Ref. ^23^), but are also detected in noncardiac/nonneurological surgery. Significant amounts of pro- and anti-inflammatory markers are detectable in plasma and CSF of older adults after knee and hip replacement surgery.^24,25^ Notably, elevation of inflammatory biomarkers has been noted after general anesthesia and spinal anesthesia.^18^ Indeed, different anesthetics may modulate immune signaling pathways (reviewed in Ref. ^26^) and perhaps cognitive outcomes.

## NEUROIMAGING

Other recent studies have used neuroimaging techniques to visualize changes in brain structure and function after surgery. Kant et al^27^ recently performed a systematic review of structural magnetic resonance imaging data in perioperative neurocognitive disorder and found a consistent association with neurovascular brain changes. The neurovascular unit is critically involved in neurodegenerative diseases and promoting brain health.^28^ The role of this interface after surgery is the focus of several ongoing preclinical studies. Initial observations using gadolinium-enhanced magnetic resonance imaging show acute (<24 hours) blood–brain barrier disruption in cardiac surgery patients, and this blood–brain barrier opening seems to correlate with subsequent neurological impairments.^29,30^ Moreover, in critically ill patients with delirium, endothelial dysfunction and impaired microvascular permeability have also been observed using peripheral artery tonometry and more recently by assessing plasma biomarkers such as S100 calcium-binding protein B, plasminogen activator inhibitor-1, and E-selectin.^31,32^ Our capacity to image neuroinflammation in humans is limited, but second-generation positron emission tomography tracers directed to the translocator protein have been developed. Although the translocator protein is upregulated by microglia after injury, other cell types and brain vessels express great affinity for the protein (given its location on the mitochondrial membrane), and thus, the proxy of the translocator protein for inflammation may be obscured by other factors.^33^ Using the ligand [^11^C]PBR28, Forsberg et al^34^ conducted the first human imaging study to evaluate neuroinflammation during the perioperative period. Although a strong immunosuppressive response was observed acutely after surgery, microglial activation was detected in a subset of patients with cognitive deficits at 3 months. The interplay between peripheral and central inflammation is a major challenge for clinical perioperative research, and more specific markers are needed to better identify immunocompetent cells using positron emission tomography.

As clinical research in this domain intensifies, fundamental questions on “how” surgery and anesthesia affect the central nervous system (CNS) warrant detailed evaluation. Establishing and refining clinically relevant surgical models to study perioperative neurocognitive disorder are contributing to a better understanding of the pathogenesis of cognitive decline and more rigorous evaluation of contributing factors such as different anesthetics, surgical procedures, genetic susceptibilities, and comorbidities. Rodents have been the primary source of preclinical data for perioperative neurocognitive disorder, and rats and mice are most commonly used to evaluate inflammatory changes and cognition after surgery. Cardiac models of surgery, recapitulating cardiopulmonary bypass and deep hypothermic circulatory arrest, have been established to assess neurological complications and perioperative inflammation.^35^ Notably, cardiac surgery triggers widespread changes in cognition that differ from abdominal surgery, while both generate substantial hippocampal neuroinflammation (ie, microglial activation).^36^ This suggests that distinct pathways may be involved in the response to injury in different organs, and further studies are needed to dissect these pathways. Abdominal and cardiac surgery were also shown to impair neuronal plasticity, as demonstrated by acute changes in hippocampal neurogenesis and brain-derived neurotrophic factor.^36^ Importantly, these changes outlasted the neuroinflammatory profile, and remained visible for weeks after surgery. Brain-derived neurotrophic factor in particular has been involved in this response and was found to be dysregulated in several other models.^37–39^ Yet, the role of inflammation in neuronal deficits and cognitive decline remains undefined, and further studies are needed. We have pioneered the development of a clinically relevant orthopedic model consisting of an intramedullary fixation of the mouse tibia.^40–42^ This has been associated with hippocampal neuroinflammation and synaptic dysfunction due to proinflammatory cytokines such as tumor necrosis factor-α, interleukin-1β, and high-mobility group box 1 protein.^43^ Models of splenectomy,^44^ hepatectomy,^45,46^ abdominal,^47,48^ and vascular surgery^49,50^ have reported hippocampal neuroinflammation and behavioral deficits. Notably, even minor surgical procedures, such as skin incisions, trigger neuroinflammation in aged animals, but not younger adults.^51^ Because aging is a critical risk factor for perioperative neurocognitive disorder, it is paramount that future research systematically evaluates advanced age, as well as sex differences and other common susceptibilities, to ensure successful translation of preclinical data to the bedside.

It is well appreciated that the nervous and immune systems bidirectionally communicate with apparent implications for both health and disease.^52,53^ Indeed, the response to peripheral surgery is able to reach the CNS via multiple pathways. Here we focus on the role of (1) systemic inflammation, (2) the neurovascular unit/blood–brain barrier, and (3) neuroinflammation after surgery.

## SYSTEMIC INFLAMMATORY RESPONSE

Systemic inflammation produces physiological and behavioral changes in humans and animals that are characterized by a decline in cognitive function, fever, decreased food intake, somnolence, hyperalgesia, and general fatigue—commonly referred to as “sickness behavior.”^54^ Sterile inflammation activates similar innate immune pathways to other stressors, such as lipopolysaccharide, by releasing damage-associated molecular patterns, such as high-mobility group box 1 protein, and cytokines.^55^ These soluble mediators can trigger a systemic inflammatory response via activation of pattern recognition receptors, including toll-like receptors, cytokines such as interleukin-1, interleukin-6, and tumor necrosis factor-α, as well as S100 Ca^2+^ binding proteins and oxidative stress pathways. We and others have shown a pivotal role for systemic alarmins and cytokines such as interleukin-1β,^40^ tumor necrosis factor-α,^41,56^ interleukin-6,^57,58^ and high-mobility group box 1 protein^43,46^ in triggering neuroinflammation after peripheral surgery in rodent models. Similar changes in biomarkers have been described in clinical samples and provide critical insights into the temporal profile of different cytokines after surgery. This should be taken into consideration as larger datasets become available for perioperative neurocognitive disorder diagnosis and treatment.

It is important to note that inflammation is overall a protective response to injury, but the improper control of its normal resolution can become harmful and contribute to pathological hallmarks including neuroinflammation.^59–61^ The impact of systemic inflammation on the brain can be profound. Mounting evidence indicates that blood-borne factors as well as the proinflammatory systemic milieu can negatively impact CNS function, directly affecting synaptic plasticity and cognitive function during normal aging.^62,63^ Uniform immune signaling responses, including monocyte activation, have also been linked to surgical trauma and may serve as predictors for different recovery profiles in at-risk patients.^64^ Peripheral cells have become attractive targets for perioperative neurocognitive disorder research because they can be easily accessed in patients and may inform subsequent changes in the CNS without accessing CSF or performing neuroimaging. Monocyte-derived macrophages migrate into the brain parenchyma after surgical trauma, and this plays a role in the pathophysiology of neurological complications including postoperative cognitive decline.^43,58,65^ In particular, it has been proposed that elevated cerebral monocyte chemoattractant protein-1 contributes to the recruitment of monocytes to the CNS and the ensuing neuroinflammatory response.^58,66^ Both tumor necrosis factor-α and high-mobility group box 1 protein have been implicated in the regulation of monocyte chemoattractant protein-1 after surgery,^41,66^ and these may serve as targets for clinical studies. Circulating monocytes, neutrophils, and other peripheral systemic factors can contribute to changes in neuronal function, synaptic plasticity, and glial homeostasis^67^; however, they are also critically involved in the release of neuroprotective factors, which is crucial in the context of surgical recovery.^68^ Overall, the contribution of other cellular factors (including T-cells and components of adaptive immunity) to perioperative neurocognitive disorder is largely understudied.

## ENDOTHELIAL DYSFUNCTION AND NEUROVASCULAR UNIT/BLOOD–BRAIN BARRIER OPENING

Endothelial cells, pericytes, and astrocytic end-feet are core components of the neurovascular unit.^69^ Together with tight junction and adherent proteins of the endothelial cell layer, they ensure proper barrier formation and protection against potentially harmful peripheral molecules.^70^ Under pathological conditions, the blood–brain barrier allows extravasation of various immune cells and systemic markers including plasma proteins, prostaglandins, cytokines, and chemokines into brain parenchyma.^71^ Surgery triggers inflammation and pattern recognition receptors expressed at the blood–brain barrier surface can lead to endothelial inflammation and subsequent neuroinflammation.^65,72–75^ This disruption is also found in several neurological disorders such as traumatic injury, stroke, and neurodegenerative diseases.^76,77^ Cytokines and migration of peripheral immunocompetent cells across the blood–brain barrier have been associated with perioperative neurocognitive disorder in animal models.^65^ After orthopedic surgery, we found opening of the blood–brain barrier with parenchymal fibrinogen deposition in the hippocampus.^65^ Using *Cx3cr1*^*GFP/+*^*Ccr2*^*RFP/+*^ transgenic mice, we described acute infiltration of monocytes C-C chemokine receptor type 2 into the brain parenchyma via processes partly mediated by tumor necrosis factor-α/nuclear factor kappa-light-chain-enhancer of activated B cells signaling in monocytes.^58,65^ Macrophage-specific deletion of IκB kinase, a central coordinator of tumor necrosis factor-α activation of nuclear factor kappa-light-chain-enhancer of activated B cells, prevented subsequent infiltration into the hippocampus after surgery. D’Mello et al^66^ described a similar immune-to-brain communication pathway after hepatic inflammation, and also demonstrated that tumor necrosis factor-α-stimulated microglia produce monocyte chemoattractant protein-1, which subsequently causes monocyte infiltration into the brain. Monocyte chemoattractant protein-1 is elevated in the CSF of a limited subset of patients with delirium after orthopedic surgery,^24^ suggesting that similar mechanisms may occur in humans. Other preclinical surgical models have found similar changes in blood–brain barrier ultrastructure, with infiltration of exogenous tracers into the brain parenchyma, as well as astrocyte pathology.^72,78^ Cardiac surgery was shown to impair expression of tight junctions in rats.^79^ Laparotomy, especially in aged mice, triggers changes in several markers including claudins, occludins, and adhesion molecules, leading to blood–brain barrier opening and cognitive decline in a process dependent on interleukin-6 signaling.^74^ Notably, other studies have shown that administration of interleukin-6 monoclonal antibody and targeting of tumor necrosis factor-α (upstream from interleukin-1 and interleukin-6 signaling) prevent perioperative neurocognitive disorder.^41,73^ Surgery was shown to upregulate enzymes that break down extracellular matrix, such as matrix metallopeptidase 9, and lead to blood–brain barrier opening and neuroinflammation.^75^ Importantly, different concentrations of sevoflurane anesthesia differentially regulate matrix metallopeptidase 9 and 2,^80^ suggesting that anesthesia per se contributes to these changes in the aging brain. Significantly more work is needed to define the role of different anesthetics in blood–brain barrier/neurovascular unit perioperative changes. Many of these pathological features, including blood–brain barrier opening, neurovascular unit dysfunction, and cell infiltration into the CNS, have been implicated in many neurological disorders.^81–83^ Yet in some cases, the infiltration of blood-derived cells, such as macrophages, is necessary to boost tissue recovery in unique ways that resident microglia, astrocytes, and oligodendrocytes cannot.^84,85^ Therefore, the role and timing of blood–brain barrier/neurovascular unit opening after surgery require further investigation to possibly develop strategies to effectively limit neuroinflammation in the perioperative period.

## NEUROINFLAMMATION

Neuroinflammation has become a key feature of virtually every neurological complication.^86,87^ Microglial activation plays a critical role in CNS dyshomeostasis.^88^ Microglia, the resident immune cells of the CNS, are highly motile cells that continuously survey the brain microenvironment, facilitating synaptic activity, pruning, and remodeling.^89,90^ Under normal conditions, microglia display highly complex and morphologies with small nuclei and slender processes.^91^ Upon injury, these cells can shift to a “reactive” phenotype, losing their ramified morphology to become enlarged and stumpy. Activated microglia have been implicated as the primary source of the CNS pro- and anti-inflammatory milieu.^92^ Activated microglia secrete proinflammatory factors such as cytokines, eicosanoids, complement factors, excitatory amino acids, reactive oxygen radicals, and nitric oxide.^93^ While dysregulation of these factors can lead to pathology, microglial activation is also responsible for jumpstarting reparative processes and releasing neuroprotective factors. For example, in Alzheimer’s disease, microglia contribute to the clearing of amyloid deposits, and support synaptic remodeling by releasing growth factors.^94^ However, microglia also contribute to pathological features in the Alzheimer’s disease brain, including hyperphosphorylation of tau and neuronal loss via cytokine release.^95^ Thus, microglia display both defensive and protective functions, making their role in neurological conditions paradoxical and poorly understood. Microglial activation has been described in several rodent models after peripheral surgery and recently, in human subjects, and is associated with longer-lasting cognitive impairments.^34^ Conventional histology is still the most common strategy for evaluating microglia in the CNS. Ionized calcium-binding adaptor molecule 1, a protein found on microglia and macrophages,^96^ is a classic marker for morphological evaluation that has often been used in perioperative neurocognitive disorder models. However, recent advancements in technology are revolutionizing our understanding of neuroinflammation in health and disease. Cell sequencing and multiomics approaches are now revealing unique phenotypes and functions of these cells during normal aging and neurodegeneration that go beyond morphological changes and immunostaining.^97–99^ Evaluation of microglia across discrete brain regions in mice has revealed selective regional sensitivity to neuroinflammation.^100^ Further, microglia isolated from different brain regions respond differently to challenges, possibly the reason that neurological disorders affect specific areas and cell populations.^101,102^ To date, a large number of studies interrogating perioperative neurocognitive disorder in rodent models have focused on the hippocampus, given its essential role in learning and memory. Within this larger framework, studies that evaluate multiple brain regions may reveal different response profiles for microglia, and possibly other cell types, related to surgery and anesthesia. Other technologies have been implemented with direct implications to neuroimmunology. Tissue clarification allows investigators to evaluate complex structures in intact specimens, including the brain.^103,104^ CLARITY is offering novel insights into 3D imaging.^105–107^ This pioneering technique preserves cellular integrity while rendering tissues visually transparent for deeper optical imaging. Our own work using CLARITY is providing ways to evaluate changes in microglial morphology after surgery and to further evaluate their relationship with other cell types including endothelial cells, neurons, and astrocytes. Indeed, microglia can induce astrocyte activation, which leads to neuronal death and toxicity.^108^ Astrocytes are also activated postoperatively in perioperative neurocognitive disorder models of major surgery (eg, liver surgery).^109^ Tibial fracture induces morphological and functional changes in astrocytes^110,111^ that contribute to the disruption of neuroglial metabolic coupling and subsequent neuronal dysfunction. Complement activation, in particular C3 and C3R, is increased in microglia and astrocytes after orthopedic surgery thereby contributing to synaptic loss and hippocampal inflammation.^112^ Similar mechanisms involved in synaptic pruning during development were described by Stevens et al,^113^ and were found to be hijacked in Alzheimer’s disease.^114^ Defining the role of complement signaling and mechanisms of communication between glia and neurons in perioperative neurocognitive disorder will require extensive studies.

The role of inflammation in perioperative brain function is becoming apparent (Figure). Although this is a necessary response to tissue trauma, defective resolution and nonresolving inflammation are now appreciated as key contributors to chronic and maladaptive states.^61^ In perioperative neurocognitive disorder, we are beginning to understand that “fine-tuning” of immune signaling may be a way forward to limit secondary CNS damage. Broad approaches that block inflammation, for example, treating with dexamethasone or statins, yield limited results in clinical trials.^115,116^ Harnessing endogenous pathways and mediators, such as cholinergic signaling and lipids biosynthesized from omega-3 fatty acids, may provide unique opportunities to curtail inflammation after surgery without causing unwanted side effects.^117^ In particular, omega-3 fatty acids are important catalysts in the synthesis of potent specialized proresolving mediators,^61^ which can exert proresolving and anti-inflammatory actions after surgery and several other conditions (reviewed in Ref. ^118^).^110,119^ Alternative approaches to regulate immunity at a neuronal level are also under development. The cholinergic anti-inflammatory reflex is one of the exemplary circuits that can regulate inflammation by stimulating the vagus nerve.^53,120,121^ The establishment of bioelectronic approaches is already able to reduce inflammation in rheumatoid arthritis patients,^122^ and may be effective in treating neuroinflammation although further research is needed.

**Figure.**
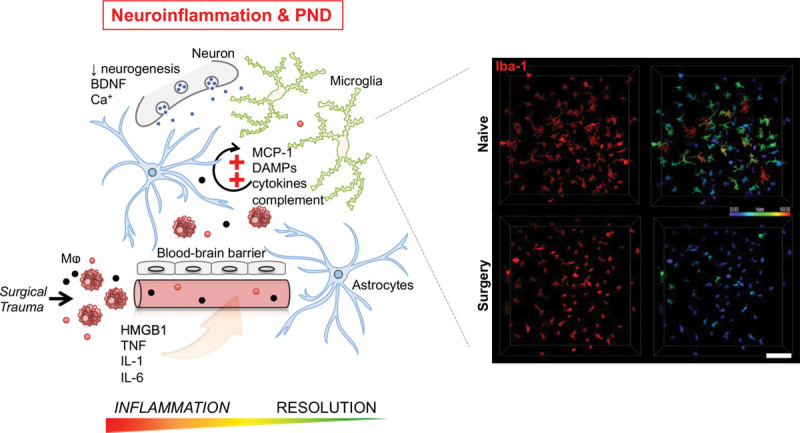
Schematic overview of mechanisms involved in postoperative neuroinflammation. Surgery activates the innate immune system resulting in release of proinflammatory mediators (cytokines, chemokines, alarmins, eicosanoids, etc) and activation of systemic immunocompetent cells. These processes negatively affect the blood–brain barrier, resulting in endothelial dysfunction and infiltration of peripheral cells/factors into the brain parenchyma. Within the central nervous system, astrocytes and microglia shift from their resting state and contribute to overall neuronal dysfunction and memory function. Harnessing resolution programs as early as the inflammatory response begins may translate into neuroprotective strategies for PND. An example of microglial activation after surgery using CLARITY is provided on the right panel. Scale bar: 150 µm. BDNF indicates brain-derived neurotrophic factor; Ca+, calcium; DAMP, damage-associated molecular pattern; HMGB1, high-mobility group box 1 protein; IL, interleukin; Mω, macrophage; MCP, monocyte chemoattractant protein; PND, perioperative neurocognitive disorder; TNF, tumor necrosis factor.

The human brain is the source of all human thought, and also the target of many neurological disorders. These disorders can cause disturbances in behavior, cognition, and emotions that can sometimes even interfere with the essence of who we are as human beings. We have an opportunity to protect our brain, at least within the perioperative space, and preserve its fundamental functions including our capacity to reason. René Descartes’ classic line from 1637 could not be more relevant for today’s research in neuroprotection: *Cogito, ergo sum*.

## ACKNOWLEDGMENTS

We thank Kathy Gage, BS (Department of Anesthesiology, Duke University Medical Center, Durham, NC) for editorial assistance. We apologize to all colleagues whose work was not cited owing to space constraints.

## DISCLOSURES

**Name:** Saraswathi Subramaniyan, PhD.

**Contribution:** This author helped write the manuscript.

**Name:** Niccolò Terrando, PhD.

**Contribution:** This author helped write and edit the manuscript.

**This manuscript was handled by:** Gregory J. Crosby, MD.

## References

[1] Evered L, Silbert B, Knopman DS (2018). Recommendations for the nomenclature of cognitive changes associated with anaesthesia and surgery-2018.. Br J Anaesth..

[2] Avelino-Silva TJ, Campora F, Curiati JA, Jacob-Filho W (2017). Association between delirium superimposed on dementia and mortality in hospitalized older adults: a prospective cohort study.. PLoS Med..

[3] Rudolph JL, Marcantonio ER (2011). Review articles: postoperative delirium: acute change with long-term implications.. Anesth Analg..

[4] American Geriatrics Society Expert Panel on Postoperative Delirium in Older Adults (2015). Postoperative delirium in older adults: best practice statement from the American Geriatrics Society.. J Am Coll Surg..

[5] Brown CH (2014). Delirium in the cardiac surgical ICU.. Curr Opin Anaesthesiol..

[6] Bruce AJ, Ritchie CW, Blizard R, Lai R, Raven P (2007). The incidence of delirium associated with orthopedic surgery: a meta-analytic review.. Int Psychogeriatr..

[7] Moller JT, Cluitmans P, Rasmussen LS (1998). Long-term postoperative cognitive dysfunction in the elderly ISPOCD1 study. ISPOCD investigators. International Study of Post-Operative Cognitive Dysfunction.. Lancet..

[8] Monk TG, Weldon BC, Garvan CW (2008). Predictors of cognitive dysfunction after major noncardiac surgery.. Anesthesiology..

[9] Evered L, Silbert B, Scott DA, Zetterberg H, Blennow K (2018). Association of changes in plasma neurofilament light and tau levels with anesthesia and surgery: results from the CAPACITY and ARCADIAN studies.. JAMA Neurol..

[10] Xie Z, Swain CA, Ward SA (2014). Preoperative cerebrospinal fluid β-amyloid/tau ratio and postoperative delirium.. Ann Clin Transl Neurol..

[11] Evered L, Silbert B, Scott DA, Ames D, Maruff P, Blennow K (2016). Cerebrospinal fluid biomarker for Alzheimer disease predicts postoperative cognitive dysfunction.. Anesthesiology..

[12] Cunningham EL, McGuinness B, McAuley DF (2018). CSF beta-amyloid 1–42 concentration predicts delirium following elective arthroplasty surgery in an observational cohort study.. Ann Surg..

[13] Anckarsäter R, Anckarsäter H, Bromander S, Blennow K, Wass C, Zetterberg H (2014). Non-neurological surgery and cerebrospinal fluid biomarkers for neuronal and astroglial integrity.. J Neural Transm (Vienna)..

[14] Tang JX, Baranov D, Hammond M, Shaw LM, Eckenhoff MF, Eckenhoff RG (2011). Human Alzheimer and inflammation biomarkers after anesthesia and surgery.. Anesthesiology..

[15] Klinger RY, James OG, Borges-Neto S, Alzheimer’s Disease Neuroimaging Initiative (ADNI) Study Group; Neurologic Outcomes Research Group (NORG) (2018). 18F-florbetapir positron emission tomography-determined cerebral β-amyloid deposition and neurocognitive performance after cardiac surgery.. Anesthesiology..

[16] Neerland BE, Hall RJ, Seljeflot I (2016). Associations between delirium and preoperative cerebrospinal fluid C-reactive protein, interleukin-6, and interleukin-6 receptor in individuals with acute hip fracture.. J Am Geriatr Soc..

[17] Westhoff D, Witlox J, Koenderman L (2013). Preoperative cerebrospinal fluid cytokine levels and the risk of postoperative delirium in elderly hip fracture patients.. J Neuroinflammation..

[18] Yeager MP, Lunt P, Arruda J, Whalen K, Rose R, DeLeo JA (1999). Cerebrospinal fluid cytokine levels after surgery with spinal or general anesthesia.. Reg Anesth Pain Med..

[19] Kato M, Suzuki H, Murakami M, Akama M, Matsukawa S, Hashimoto Y (1997). Elevated plasma levels of interleukin-6, interleukin-8, and granulocyte colony-stimulating factor during and after major abdominal surgery.. J Clin Anesth..

[20] Bromander S, Anckarsäter R, Kristiansson M (2012). Changes in serum and cerebrospinal fluid cytokines in response to non-neurological surgery: an observational study.. J Neuroinflammation..

[21] Kálmán J, Juhász A, Bogáts G (2006). Elevated levels of inflammatory biomarkers in the cerebrospinal fluid after coronary artery bypass surgery are predictors of cognitive decline.. Neurochem Int..

[22] Ramlawi B, Rudolph JL, Mieno S (2006). C-Reactive protein and inflammatory response associated to neurocognitive decline following cardiac surgery.. Surgery..

[23] Berger M, Terrando N, Smith SK, Browndyke JN, Newman MF, Mathew JP (2018). Neurocognitive function after cardiac surgery: from phenotypes to mechanisms.. Anesthesiology..

[24] Hirsch J, Vacas S, Terrando N (2016). Perioperative cerebrospinal fluid and plasma inflammatory markers after orthopedic surgery.. J Neuroinflammation..

[25] Buvanendran A, Kroin JS, Berger RA (2006). Upregulation of prostaglandin E2 and interleukins in the central nervous system and peripheral tissue during and after surgery in humans.. Anesthesiology..

[26] Yuki K, Eckenhoff RG (2016). Mechanisms of the immunological effects of volatile anesthetics: a review.. Anesth Analg..

[27] Kant IMJ, de Bresser J, van Montfort SJT, Slooter AJC, Hendrikse J (2017). MRI markers of neurodegenerative and neurovascular changes in relation to postoperative delirium and postoperative cognitive decline.. Am J Geriatr Psychiatry..

[28] Iadecola C (2017). The neurovascular unit coming of age: a journey through neurovascular coupling in health and disease.. Neuron..

[29] Merino JG, Latour LL, Tso A (2013). Blood-brain barrier disruption after cardiac surgery.. AJNR Am J Neuroradiol..

[30] Abrahamov D, Levran O, Naparstek S (2017). Blood-brain barrier disruption after cardiopulmonary bypass: diagnosis and correlation to cognition.. Ann Thorac Surg..

[31] Hughes CG, Morandi A, Girard TD (2013). Association between endothelial dysfunction and acute brain dysfunction during critical illness.. Anesthesiology..

[32] Hughes CG, Pandharipande PP, Thompson JL (2016). Endothelial activation and blood-brain barrier injury as risk factors for delirium in critically ill patients.. Crit Care Med..

[33] Veronese M, Reis Marques T, Bloomfield PS (2018). Kinetic modelling of [11C]PBR28 for 18 kDa translocator protein PET data: a validation study of vascular modelling in the brain using XBD173 and tissue analysis.. J Cereb Blood Flow Metab..

[34] Forsberg A, Cervenka S, Jonsson Fagerlund M (2017). The immune response of the human brain to abdominal surgery.. Ann Neurol..

[35] Zhang Z, Ma Q, Shah B (2017). Neuroprotective effects of annexin A1 tripeptide after deep hypothermic circulatory arrest in rats.. Front Immunol..

[36] Hovens IB, van Leeuwen BL, Mariani MA, Kraneveld AD, Schoemaker RG (2016). Postoperative cognitive dysfunction and neuroinflammation; cardiac surgery and abdominal surgery are not the same.. Brain Behav Immun..

[37] Fidalgo AR, Cibelli M, White JP (2011). Peripheral orthopaedic surgery down-regulates hippocampal brain-derived neurotrophic factor and impairs remote memory in mouse.. Neuroscience..

[38] Tian XS, Tong YW, Li ZQ (2015). Surgical stress induces brain-derived neurotrophic factor reduction and postoperative cognitive dysfunction via glucocorticoid receptor phosphorylation in aged mice.. CNS Neurosci Ther..

[39] Zhang MD, Barde S, Yang T (2016). Orthopedic surgery modulates neuropeptides and BDNF expression at the spinal and hippocampal levels.. Proc Natl Acad Sci U S A..

[40] Cibelli M, Fidalgo AR, Terrando N (2010). Role of interleukin-1beta in postoperative cognitive dysfunction.. Ann Neurol..

[41] Terrando N, Monaco C, Ma D, Foxwell BM, Feldmann M, Maze M (2010). Tumor necrosis factor-alpha triggers a cytokine cascade yielding postoperative cognitive decline.. Proc Natl Acad Sci U S A..

[42] Xiong C, Zhang Z, Baht GS, Terrando N (2018 February). A mouse model of orthopedic surgery to study postoperative cognitive dysfunction and tissue regeneration.. J Vis Exp.

[43] Vacas S, Degos V, Tracey KJ, Maze M (2014). High-mobility group box 1 protein initiates postoperative cognitive decline by engaging bone marrow-derived macrophages.. Anesthesiology..

[44] Wan Y, Xu J, Ma D, Zeng Y, Cibelli M, Maze M (2007). Postoperative impairment of cognitive function in rats: a possible role for cytokine-mediated inflammation in the hippocampus.. Anesthesiology..

[45] Cao XZ, Ma H, Wang JK (2010). Postoperative cognitive deficits and neuroinflammation in the hippocampus triggered by surgical trauma are exacerbated in aged rats.. Prog Neuropsychopharmacol Biol Psychiatry..

[46] Terrando N, Yang T, Wang X (2016). Systemic HMGB1 neutralization prevents postoperative neurocognitive dysfunction in aged rats.. Front Immunol..

[47] Hovens IB, van Leeuwen BL, Nyakas C, Heineman E, van der Zee EA, Schoemaker RG (2015). Postoperative cognitive dysfunction and microglial activation in associated brain regions in old rats.. Neurobiol Learn Mem..

[48] Xu Z, Dong Y, Wang H (2014). Age-dependent postoperative cognitive impairment and Alzheimer-related neuropathology in mice.. Sci Rep..

[49] Zheng B, Lai R, Li J, Zuo Z (2017). Critical role of P2X7 receptors in the neuroinflammation and cognitive dysfunction after surgery.. Brain Behav Immun..

[50] Zhang J, Tan H, Jiang W, Zuo Z (2015). The choice of general anesthetics may not affect neuroinflammation and impairment of learning and memory after surgery in elderly rats.. J Neuroimmune Pharmacol..

[51] Rosczyk HA, Sparkman NL, Johnson RW (2008). Neuroinflammation and cognitive function in aged mice following minor surgery.. Exp Gerontol..

[52] Terrando N, Pavlov VA (2018). Editorial: neuro-immune interactions in inflammation and autoimmunity.. Front Immunol..

[53] Pavlov VA, Tracey KJ (2017). Neural regulation of immunity: molecular mechanisms and clinical translation.. Nat Neurosci..

[54] Dantzer R (2004). Cytokine-induced sickness behaviour: a neuroimmune response to activation of innate immunity.. Eur J Pharmacol..

[55] Lord JM, Midwinter MJ, Chen YF (2014). The systemic immune response to trauma: an overview of pathophysiology and treatment.. Lancet..

[56] Culley DJ, Snayd M, Baxter MG (2014). Systemic inflammation impairs attention and cognitive flexibility but not associative learning in aged rats: possible implications for delirium.. Front Aging Neurosci..

[57] Hovens IB, van Leeuwen BL, Nyakas C, Heineman E, van der Zee EA, Schoemaker RG (2015). Prior infection exacerbates postoperative cognitive dysfunction in aged rats.. Am J Physiol Regul Integr Comp Physiol..

[58] Degos V, Vacas S, Han Z (2013). Depletion of bone marrow-derived macrophages perturbs the innate immune response to surgery and reduces postoperative memory dysfunction.. Anesthesiology..

[59] Steinman L (2013). Inflammatory cytokines at the summits of pathological signal cascades in brain diseases.. Sci Signal..

[60] Mrak RE, Griffin WS (2005). Glia and their cytokines in progression of neurodegeneration.. Neurobiol Aging..

[61] Nathan C, Ding A (2010). Nonresolving inflammation.. Cell..

[62] Villeda SA, Luo J, Mosher KI (2011). The ageing systemic milieu negatively regulates neurogenesis and cognitive function.. Nature..

[63] Villeda SA, Plambeck KE, Middeldorp J (2014). Young blood reverses age-related impairments in cognitive function and synaptic plasticity in mice.. Nat Med..

[64] Gaudillière B, Fragiadakis GK, Bruggner RV (2014). Clinical recovery from surgery correlates with single-cell immune signatures.. Sci Transl Med..

[65] Terrando N, Eriksson LI, Ryu JK (2011). Resolving postoperative neuroinflammation and cognitive decline.. Ann Neurol..

[66] D’Mello C, Le T, Swain MG (2009). Cerebral microglia recruit monocytes into the brain in response to tumor necrosis factoralpha signaling during peripheral organ inflammation.. J Neurosci..

[67] Perry VH, Newman TA, Cunningham C (2003). The impact of systemic infection on the progression of neurodegenerative disease.. Nat Rev Neurosci..

[68] Reyes TM, Fabry Z, Coe CL (1999). Brain endothelial cell production of a neuroprotective cytokine, interleukin-6, in response to noxious stimuli.. Brain Res..

[69] Abbott NJ, Rönnbäck L, Hansson E (2006). Astrocyte-endothelial interactions at the blood-brain barrier.. Nat Rev Neurosci..

[70] Abbott NJ, Patabendige AA, Dolman DE, Yusof SR, Begley DJ (2010). Structure and function of the blood-brain barrier.. Neurobiol Dis..

[71] Daneman R (2012). The blood-brain barrier in health and disease.. Ann Neurol..

[72] He HJ, Wang Y, Le Y (2012). Surgery upregulates high mobility group box-1 and disrupts the blood-brain barrier causing cognitive dysfunction in aged rats.. CNS Neurosci Ther..

[73] Hu J, Feng X, Valdearcos M (2018). Interleukin-6 is both necessary and sufficient to produce perioperative neurocognitive disorder in mice.. Br J Anaesth..

[74] Yang S, Gu C, Mandeville ET (2017). Anesthesia and surgery impair blood-brain barrier and cognitive function in mice.. Front Immunol..

[75] Bi J, Shan W, Luo A, Zuo Z (2017). Critical role of matrix metallopeptidase 9 in postoperative cognitive dysfunction and age-dependent cognitive decline.. Oncotarget..

[76] Huber JD, Egleton RD, Davis TP (2001). Molecular physiology and pathophysiology of tight junctions in the blood-brain barrier.. Trends Neurosci..

[77] Persidsky Y, Ramirez SH, Haorah J, Kanmogne GD (2006). Blood-brain barrier: structural components and function under physiologic and pathologic conditions.. J Neuroimmune Pharmacol..

[78] Li Z, Mo N, Li L (2016). Surgery-induced hippocampal angiotensin II elevation causes blood-brain barrier disruption via MMP/TIMP in aged rats.. Front Cell Neurosci..

[79] Bartels K, Ma Q, Venkatraman TN (2014). Effects of deep hypothermic circulatory arrest on the blood brain barrier in a cardiopulmonary bypass model–a pilot study.. Heart Lung Circ..

[80] Hu N, Guo D, Wang H (2014). Involvement of the blood-brain barrier opening in cognitive decline in aged rats following orthopedic surgery and high concentration of sevoflurane inhalation.. Brain Res..

[81] Morganti JM, Jopson TD, Liu S (2015). CCR2 antagonism alters brain macrophage polarization and ameliorates cognitive dysfunction induced by traumatic brain injury.. J Neurosci..

[82] Yamasaki R, Lu H, Butovsky O (2014). Differential roles of microglia and monocytes in the inflamed central nervous system.. J Exp Med..

[83] Varvel NH, Neher JJ, Bosch A (2016). Infiltrating monocytes promote brain inflammation and exacerbate neuronal damage after status epilepticus.. Proc Natl Acad Sci U S A..

[84] Shechter R, London A, Varol C (2009). Infiltrating blood-derived macrophages are vital cells playing an anti-inflammatory role in recovery from spinal cord injury in mice.. PLoS Med..

[85] Wattananit S, Tornero D, Graubardt N (2016). Monocyte-derived macrophages contribute to spontaneous long-term functional recovery after stroke in mice.. J Neurosci..

[86] Heneka MT, Carson MJ, El Khoury J (2015). Neuroinflammation in Alzheimer’s disease.. Lancet Neurol..

[87] Wyss-Coray T (2016). Ageing, neurodegeneration and brain rejuvenation.. Nature..

[88] Ransohoff RM, El Khoury J (2015). Microglia in health and disease.. Cold Spring Harb Perspect Biol..

[89] Paolicelli RC, Gross CT (2011). Microglia in development: linking brain wiring to brain environment.. Neuron Glia Biol..

[90] Colonna M, Butovsky O (2017). Microglia function in the central nervous system during health and neurodegeneration.. Annu Rev Immunol..

[91] Nimmerjahn A, Kirchhoff F, Helmchen F (2005). Resting microglial cells are highly dynamic surveillants of brain parenchyma in vivo.. Science..

[92] Hanisch UK (2002). Microglia as a source and target of cytokines.. Glia..

[93] Minagar A, Shapshak P, Fujimura R, Ownby R, Heyes M, Eisdorfer C (2002). The role of macrophage/microglia and astrocytes in the pathogenesis of three neurologic disorders: HIV-associated dementia, Alzheimer disease, and multiple sclerosis.. J Neurol Sci..

[94] Weinhard L, di Bartolomei G, Bolasco G (2018). Microglia remodel synapses by presynaptic trogocytosis and spine head filopodia induction.. Nat Commun..

[95] Maphis N, Xu G, Kokiko-Cochran ON (2015). Reactive microglia drive tau pathology and contribute to the spreading of pathological tau in the brain.. Brain..

[96] Ahmed Z, Shaw G, Sharma VP, Yang C, McGowan E, Dickson DW (2007). Actin-binding proteins coronin-1a and IBA-1 are effective microglial markers for immunohistochemistry.. J Histochem Cytochem..

[97] Olah M, Patrick E, Villani AC (2018). A transcriptomic atlas of aged human microglia.. Nat Commun..

[98] Hickman SE, Kingery ND, Ohsumi TK (2013). The microglial sensome revealed by direct RNA sequencing.. Nat Neurosci..

[99] Galatro TF, Holtman IR, Lerario AM (2017). Transcriptomic analysis of purified human cortical microglia reveals age-associated changes.. Nat Neurosci..

[100] Grabert K, Michoel T, Karavolos MH (2016). Microglial brain region-dependent diversity and selective regional sensitivities to aging.. Nat Neurosci..

[101] Kostuk EW, Cai J, Iacovitti L (2018). Regional microglia are transcriptionally distinct but similarly exacerbate neurodegeneration in a culture model of Parkinson’s disease.. J Neuroinflammation..

[102] De Biase LM, Schuebel KE, Fusfeld ZH (2017). Local cues establish and maintain region-specific phenotypes of basal ganglia microglia.. Neuron..

[103] Chung K, Wallace J, Kim SY (2013). Structural and molecular interrogation of intact biological systems.. Nature..

[104] Chung K, Deisseroth K (2013). CLARITY for mapping the nervous system.. Nat Methods..

[105] Hsueh B, Burns VM, Pauerstein P (2017). Pathways to clinical CLARITY: volumetric analysis of irregular, soft, and heterogeneous tissues in development and disease.. Sci Rep..

[106] Epp JR, Niibori Y, Liz Hsiang HL (2015 May 25). Optimization of CLARITY for clearing whole-brain and other intact organs(1,2,3).. eNeuro..

[107] Yang B, Treweek JB, Kulkarni RP (2014). Single-cell phenotyping within transparent intact tissue through whole-body clearing.. Cell..

[108] Liddelow SA, Guttenplan KA, Clarke LE (2017). Neurotoxic reactive astrocytes are induced by activated microglia.. Nature..

[109] Wan Y, Xu J, Meng F (2010). Cognitive decline following major surgery is associated with gliosis, β-amyloid accumulation, and τ phosphorylation in old mice.. Crit Care Med..

[110] Terrando N, Gómez-Galán M, Yang T (2013). Aspirin-triggered resolvin D1 prevents surgery-induced cognitive decline.. FASEB J..

[111] Femenía T, Giménez-Cassina A, Codeluppi S (2018). Disrupted neuroglial metabolic coupling after peripheral surgery.. J Neurosci..

[112] Xiong C, Liu J, Lin D, Zhang J, Terrando N, Wu A (2018). Complement activation contributes to perioperative neurocognitive disorders in mice.. J Neuroinflammation..

[113] Stevens B, Allen NJ, Vazquez LE (2007). The classical complement cascade mediates CNS synapse elimination.. Cell..

[114] Hong S, Beja-Glasser VF, Nfonoyim BM (2016). Complement and microglia mediate early synapse loss in Alzheimer mouse models.. Science..

[115] Ottens TH, Dieleman JM, Sauër AM, DExamethasone for Cardiac Surgery (DECS) Study Group (2014). Effects of dexamethasone on cognitive decline after cardiac surgery: a randomized clinical trial.. Anesthesiology..

[116] Page VJ, Casarin A, Ely EW (2017). Evaluation of early administration of simvastatin in the prevention and treatment of delirium in critically ill patients undergoing mechanical ventilation (MoDUS): a randomised, double-blind, placebo-controlled trial.. Lancet Respir Med..

[117] Serhan CN (2017). Treating inflammation and infection in the 21st century: new hints from decoding resolution mediators and mechanisms.. FASEB J..

[118] Serhan CN, Levy BD (2018). Resolvins in inflammation: emergence of the pro-resolving superfamily of mediators.. J Clin Invest..

[119] Feng X, Valdearcos M, Uchida Y, Lutrin D, Maze M, Koliwad SK (2017). Microglia mediate postoperative hippocampal inflammation and cognitive decline in mice.. JCI Insight..

[120] Steinberg BE, Sundman E, Terrando N, Eriksson LI, Olofsson PS (2016). Neural control of inflammation: implications for perioperative and critical care.. Anesthesiology..

[121] Huffman WJ, Subramaniyan S, Rodriguiz RM, Wetsel WC, Grill WM, Terrando N (2019). Modulation of neuroinflammation and memory dysfunction using percutaneous vagus nerve stimulation in mice.. Brain Stimul..

[122] Koopman FA, Chavan SS, Miljko S (2016). Vagus nerve stimulation inhibits cytokine production and attenuates disease severity in rheumatoid arthritis.. Proc Natl Acad Sci U S A..

